# Dynamic functional and structural remodeling during retinal regeneration in zebrafish

**DOI:** 10.3389/fnmol.2022.1070509

**Published:** 2022-11-30

**Authors:** Lindsey M. Barrett, Diana M. Mitchell, Peter C. Meighan, Michael D. Varnum, Deborah L. Stenkamp

**Affiliations:** ^1^Department of Biological Sciences, University of Idaho, Moscow, ID, United States; ^2^Department of Integrative Physiology and Neuroscience, Washington State University, Pullman, WA, United States

**Keywords:** retina, regeneration, zebrafish, electroretinography, retinal bipolar cell, ERG, ON-bipolar, OFF-bipolar

## Abstract

**Introduction:**

Zebrafish regenerate their retinas following damage, resulting in restoration of visual function. Here we evaluate recovery of retinal function through qualitative and quantitative analysis of the electroretinogram (ERG) over time following retinal damage, in correlation to histological features of regenerated retinal tissue.

**Methods:**

Retinas of adult zebrafish were lesioned by intravitreal injection of 10 μM (extensive lesion; destroys all neurons) or 2 μM (selective lesion; spares photoreceptors) ouabain. Unlesioned contralateral retinas served as controls. Function of retinal circuitry was analyzed at selected timepoints using ERG recordings from live zebrafish, and whole eyes were processed for histological analyses immediately thereafter.

**Results:**

Qualitative and quantitative assessment of waveforms during retinal regeneration revealed dynamic changes that were heterogeneous on an individual level within each sampling time, but still followed common waveform recovery patterns on a per-fish and population-level basis. Early in the regeneration period (13–30 days post injury; DPI), for both lesion types, b-waves were essentially not detected, and unmasked increased apparent amplitudes, implicit times, and half-widths of a-waves (vs. controls). In control recordings, d-waves were not obviously detected, but apparent d-waves (OFF-bipolar responses) from regenerating retinas of several fish became prominent by 30DPI and dominated the post-photoreceptor response (PPR). Beyond 45DPI, b-waves became detectable, and the ratio of apparent d- to b-wave contributions progressively shifted with most, but not all, fish displaying a b-wave dominated PPR. At the latest timepoints (extensive, 90DPI; selective, 80DPI), recordings with measurable b-waves approached a normal waveform (implicit times and half-widths), but amplitudes were not restored to control levels. Histological analyses of the retinas from which ERGs were recorded showed that as regeneration progressed, PKCa + ON-bipolar terminals and parvalbumin + amacrine cell processes became more stereotypically positioned within the deep sublaminae of the INL over recovery time after each lesion type, consistent with the shift in PPR seen in the ERG recordings.

**Discussion:**

Taken together, these data suggest that photoreceptor-OFF-bipolar component/connectivity may functionally recover and mature earlier during regeneration compared to the photoreceptor-ON-bipolar component, though the timeframe in which such recovery happens is heterogeneous on a per-fish basis. Collectively our studies suggest gradual restoration of ON-bipolar functional circuitry during retinal regeneration.

## Introduction

The vertebrate neural retina is a laminated structure of the central nervous system, with specialized circuitry to accomplish not simply light detection, but also contrast enhancement, edge detection, color processing, and motion and feature extraction (Dowling, 1987). These and other embedded functions of the retina have presented obstacles to the production of retinal prosthetics in the treatment of human retinal damage and disease (Ayton et al., 2020). Furthermore, the damaged mammalian retina undergoes a remodeling process that presents difficulties in integrating a prosthesis into existing circuitry (Dagnelie, 2012). Alternative approaches for treating retinal damage and degeneration, such as the transplantation of laboratory-grown retinal organoids, or the induction of a retinal regenerative process, face similar obstacles. Will the transplanted or regenerated neurons establish the correct connections with each other and with any existing neurons that survived the damage/disorders (Angueyra and Kindt, 2018)? Will these neurons function adequately to sufficiently restore vision? Recently we and others have investigated these experimental questions in the zebrafish model, in which retinal regeneration takes place endogenously in response to the loss of neurons. In larval (D’Orazi et al., 2016) and adult (McGinn et al., 2018, 2019) zebrafish, regenerated retinal bipolar neurons re-establish microanatomical connections with undamaged cone photoreceptors, with very little evidence of error. Electroretinogram (ERG; a sum field potential of the retina in response to a light stimulus) recordings of regenerated adult zebrafish retina showed a normal-appearing waveform that, although reduced in amplitude, is consistent with accurate connectivity (McGinn et al., 2018).

The restoration of synaptic connectivity and physiological responses in regenerated adult zebrafish retina (McGinn et al., 2018) is even more remarkable considering that the initial damage was a tissue-disrupting chemical lesion that destroyed inner retinal neurons (ganglion cells, amacrine cells, bipolar cells, and most horizontal cells) but spared photoreceptors and glia (Fimbel et al., 2007; Sherpa et al., 2014; McGinn et al., 2018). Such an injury generates widespread cell death and an accumulation of leukocytes (Mitchell et al., 2018), and triggers Müller glia to re-enter the cell cycle and undergo an asymmetric cell division to generate retinal progenitors (Nagashima et al., 2013). These progenitors repopulate the retina with regenerated neurons, but the process is distinct from embryonic development in that the birth order of ganglion cells and bipolar cells is overlapping rather than sequential (McGinn et al., 2019), and there are errors in lamination such that neuronal cell bodies occupy the inner plexiform (synaptic) layer (IPL), particularly early in the regeneration process (Fimbel et al., 2007; Sherpa et al., 2014). Many of these regenerative responses would appear to set the stage for connectivity errors or delays in functional recovery, and yet the regenerated zebrafish retina functions (McGinn et al., 2018) and supports visually-mediated behaviors (Sherpa et al., 2014). Interestingly, regeneration of bipolar neurons may occur in a protracted process (McGinn et al., 2019); whether functional recovery occurs similarly, or whether/when distinct electrical signals emerge over time, is unknown.

In the present study, we document a time-course of retinal regeneration in adult zebrafish, using retinal function, as revealed by ERG recordings, as the primary experimental endpoint. We also correlate the analysis of ERG recordings to the reappearance of regenerated neurons and synaptic processes. Major ERG waveform components were identified by polarity and timing with respect to the light stimulus. Our purpose was to gain insights into the process of functional recovery in correlation to histological regeneration and to identify any aberrant physiological responses. For example, is functional wiring recovered gradually over time (as shown for visually-mediated reflexes and behaviors following a light lesion (Hammer et al., 2021)) or does it re-appear suddenly at a certain time window? Does the overall shape of the waveform change over time? For this study, we evaluated regeneration after lesions to the inner retina, described in the preceding paragraph, and after more extensive chemical lesions that destroy all retinal neurons and spare only glia (Sherpa et al., 2008). For histological correlation, we examined the patterns of expression of selected cell-specific and synaptic markers in retinal tissue, including those that characterize the IPL within the retinas from which ERGs were recorded. We find considerable heterogeneity in ERG waveforms recorded at selected timepoints, including the fraction of retinas with measurable waveform components, suggesting asynchrony in regeneration among individual fish. Interestingly, a post-photoreceptor response (PPR) component of the ERG, likely representing bipolar cell function, is detected in many individuals early (13 days post-injury; DPI) after both lesion types. This early-emerging PPR is evident at the end of the light flash, and can be isolated with a longer light pulse, consistent with dominance of an apparent d-wave - the response of OFF-bipolar neurons – at this stage of regeneration. Over time, the PPR begins to shift to a waveform that is consistent with the emergence of a stronger b-wave contribution, representing the response of ON-bipolar neurons, at 45-90 DPI for an extensive lesion, and at 30-80 DPI for an inner retina-selective lesion. The patterns of expression of markers of the ON-bipolar sublaminae of the IPL were consistent with these physiological findings. We consider that the changes in ERG waveform over time probably reflect changing net balance of contributing depolarizating and hyperpolarizing signals, plus potential inhibitory inputs, of existing and regenerating neurons. Our studies are the first to illuminate the time-course of functional recovery of photoreceptor-bipolar circuitry in regenerating zebrafish retina. They suggest that functional restoration of vision is a temporally heterogenous process on an individual basis; however, it generally follows a pattern in which OFF-bipolar circuitry is restored earlier than ON-bipolar circuitry during retinal regeneration.

## Materials and methods

### Animals

Zebrafish (*Danio rerio*) were raised and maintained according to Westerfield (2007) in monitored, recirculating system water, on a 14:10 h light:dark cycle. The transgenic strain *sws2:mCherry*, in which mCherry is expressed in SWS2 (blue-sensitive) cones (Salbreux et al., 2012), was the kind gift of Dr. Pamela Raymond, and the transgenic strain *nyx:mYFP* (two transgenes likely cointegrated), in which the *nyctalopin* (*nyx*) promoter drives gal4 and the *UAS* enhancer element drives MYFP, and which shows variegated expression of YFP in a subpopulation of retinal bipolar neurons (Schroeter et al., 2006), was the kind gift of Dr. Rachel Wong. Zebrafish of both sexes were used for experiments. Animals were treated in accordance with protocols approved by the University of Idaho and Washington State University Institutional Animal Care and Use Committees.

### Retinal lesioning and verification

The retinas of adult fish (6–16 months old; both sexes) were chemically lesioned as previously described (Maier and Wolburg, 1979; Raymond et al., 1988; Fimbel et al., 2007; Sherpa et al., 2008, 2014; Nagashima et al., 2013; McGinn et al., 2018, 2019; Mitchell et al., 2018, 2019; Mitchell and Stenkamp, 2022). Retinal damage was achieved by intraocular injection of the neurotoxin ouabain (Sigma), either to destroy all neurons but spare glia (“extensive” lesion; (Sherpa et al., 2008)), or to destroy inner retinal neurons but spare glia and photoreceptors (“selective” lesion; (Fimbel et al., 2007; Nagashima et al., 2013; Sherpa et al., 2014; McGinn et al., 2018, 2019; Mitchell et al., 2018, 2019; Mitchell and Stenkamp, 2022)). Working stocks of 40 μM (for selective lesion) and 200 μM (for extensive lesion) ouabain octahydrate were prepared in 0.65% sterile saline (NaCl). Fish were anesthetized by immersion in buffered (pH 7.0-7.4) tricaine methanesulfonate (MS-222; Pentair Aquatic Ecosystems) (Westerfield, 2007), and corneas were perforated with a sapphire blade. A Hamilton syringe (26 s) was introduced through the incision and used to inject 0.4-0.6 μL of the ouabain solution into the vitreal chamber. The specific volume injected was calculated based on eye diameter (measured with calipers) and solid geometries of the eye and lens, to result in an estimated final intraocular concentration of 10-20 μM (extensive lesion) and 2-4 μM (selective lesion). Only right eyes were injected, with the left eyes serving as undamaged controls.

Loss of retinal neurons including photoreceptors in extensively lesioned fish was verified by viewing retinas of live, anesthetized *sws2:mCherry* fish with epifluorescence stereomicroscopy (Nikon SMZ 1500) at 3 or 4 days post injury (DPI) for the absence of mCherry in the lesioned eye, and the presence of the reporter in the contralateral eye (Mitchell and Stenkamp, 2022). In addition, a subset of extensively lesioned zebrafish was processed for cryosectioning of eyes and confocal imaging at 3 or 4 DPI (see *Imaging* below). Similarly, verification of loss of neurons but survival of photoreceptors in selectively lesioned fish was done by viewing retinas of live, anesthetized *sws2:mCherry; nyx:mYFP* fish for the absence of YFP but presence of mCherry in the lesioned eye at 3 DPI (Mitchell and Stenkamp, 2022), and by imaging cryosections of a subset of selectively lesioned fish processed at 3 DPI (McGinn et al., 2018, 2019).

### Electroretinogram (ERG) recordings of damaged and regenerated retinas

ERG recordings and analysis were similar to the methods used in McGinn et al. (2018) and described in more detail in (Barrett et al., 2022). Custom ERG instrumentation (Barrett et al., 2022) was built and assembled based upon that of (Makhankov et al., 2004). Adult zebrafish were subjected to approximately 2-3 hours of dim light adaptation, and then anesthetized in buffered 200 mg/L MS-222 containing 0.1 mg/mL rorcuronium bromide (a neuromuscular blocker). A tube was inserted into the fish’s oral cavity to supply a continuous (1 mL/min) source of this solution, which was aerated, for the entirety of the procedure. All ERG measurements were performed in the early afternoon, from 12:00–17:00. A Solis-3C High-Power White LED (Thorlabs) served as the light source. Maximum light intensity was 5000 lux. Light intensity was controlled through neutral-density filters with defined log unit attenuation. Data were averaged (7–8 responses to a 200 ms flash, with a 10 s ISI), digitized, and analyzed using a computer interfaced PowerLab 200 (AD Instruments) and GraphPad Prism software. Data were sampled at a rate of 1.0 kHz and differentially amplified 1000 × with a band-pass filter between 0.1 and 500 Hz. Grand average waveforms were generated to represent responses of undamaged control retinas, damaged retinas, and regenerating/regenerated retinas at selected analysis times for extensively lesioned (4, 13, 21, 30, 45, 60, and 90/94 DPI) and selectively lesioned (3, 13, 21, 30, and 80 DPI) zebrafish. To facilitate qualitative comparisons across diverse ERG responses during regeneration, grand average waveforms also were subsequently scaled to normalize the range of extreme values (positive and negative voltage deflections from baseline) such that the amplitude range for each grand average was set at 100% (maximum) to 0% (minimum).

### Electroretinogram (ERG) analyses

We focused our qualitative analysis upon the “post-photoreceptor response” (PPR; upward or depolarizing deflection of the waveform) through comparison of waveforms over time after lesioning. Nearly all recordings were included in the assembly of grand average waveforms, including those with little or no response to the light flash (n = 3-7 recordings were used for each sampling time; the 90 and 94 DPI recordings for extensive lesion were combined). Exclusionary criteria were i) recordings in which opercular or other movements persisted and interfered with the recordings (Barrett et al., 2022); ii) recordings from 3 or 4 DPI retinas which, upon histological inspection, did not display indications of damage (i.e., no loss of neurons); and iii) recordings from regenerating/regenerated retinas which, upon histological inspection, did not display indications of regeneration (i.e., no cell bodies misplaced in plexiform layers, also known as laminar fusions; (Hitchcock and Raymond, 1992; Cameron and Carney, 2004; Sherpa et al., 2014)).

To quantify and further characterize features of putative a-waves and PPRs, we established the following criteria for identification of a-waves, b-waves, and d-waves: 1) Any downward deflection (hyperpolarizing response) evident during the light stimulus (0-200 ms) and having a peak greater than 15 μv below baseline was considered an a-wave. 2) Any upward deflection (depolarizing response) during the light stimulus (0-200 ms) and having a peak greater than 15 μv above baseline was considered a b-wave. 3) Any upward deflection rising after light offset (200-500 ms) and having a peak greater than 15 μv above baseline was considered a d-wave. Furthermore, longer duration light stimulus protocols were used to confirm that the apparent OFF-BP responses (d-wave) segregated from other possible contributions. Supplementary Figure 1 shows examples of raw traces displaying an a-wave only (Supplementary Figure 1), a- and b-waves (Supplementary Figure 1), a- and d-waves (Supplementary Figure 1), and a-, b-, and d-waves (Supplementary Figure 1). Peak amplitudes for each identified wave were measured as the greatest deflection from baseline for that peak (Supplementary Figure 1). Implicit times for each wave were measured as the time from stimulus onset (for a- and b-waves) or offset (for d-waves) to the time of the peak for that particular wave (Supplementary Figure 1), and “half-widths” for each wave were measured as the width of the wave at half-maximal amplitude (Supplementary Figure 1). Across the time-course series for each measurement (amplitude, implicit time, half-width), statistical comparisons were made using Kruskal-Wallis tests followed by *post hoc* tests of the Dunn method, with Benjamini-Hochberg FDR adjustment, where justified. Statistical analysis of proportions of no-response values were performed by using a generalized linear model with ANOVA, with *post hoc* pairwise proportion test. Results of statistical analyses are shown in Supplementary Tables 1–8.

### Tissue processing and immunohistochemistry

Immediately following ERG recordings, tissue samples were collected for analysis. Whole eyes were enucleated with fine forceps, and the lens was removed. Eyes were fixed in phosphate-buffered (pH 7.4) 5% sucrose containing 4% paraformaldehyde overnight at 4°C, and then washed in a graded sucrose series ending in a 20% sucrose cryoprotection step overnight at 4°C. Eyes were embedded in a 1:2 solution of optimal cutting temperature (OCT) embedding medium (Sakura Finetek) and phosphate-buffered, 20% sucrose, and frozen in liquid N_2_-cooled isobutane. Eyes were sectioned at 5 μm on a Leica CM3050 cryostat. Tissue sections were placed on glass slides and desiccated overnight, then stored at −20°C until staining.

Non-specific immunolabeling was blocked by including 20% goat serum in PBS containing 0.5% Triton X-100 (Sigma) (PBST) at room temperature for 30 min, incubated with primary antibody in PBST with 1% goat serum overnight at 4°C, washed in PBST, incubated in secondary antibody in PBST with 1% goat serum plus 0.5 μg/mL DAPI for 30 minutes at room temperature, and washed again in PBST prior to mounting with Vectashield Vibrance (Vector Labs) and coverslipping.

Primary antibodies used in this study were: anti-protein kinase Cα (PKCα), a mouse monoclonal antibody that labels a subpopulation of retinal bipolar neurons (Suzuki and Kaneko, 1990) (Santa Cruz Biotech PKC Antibody (A-3), #sc-17769, RRID:AB_628139; 1:200); anti-synaptic vesicle 2 (SV2), a mouse monoclonal antibody that labels synaptic terminals (Yazulla and Studholme, 2001) (Developmental Studies Hybridoma Bank SV2-s, RRID:AB_2315387; 1:2000); and anti-parvalbumin (PARV), a mouse monoclonal that labels a subpopulation of amacrine cells and their processes in the inner plexiform layer (IPL) (Yeo et al., 2009) (Thermofisher #MAB1572MI, RRID:AB_2174013; 1:1,000). Secondary antibodies used in this study were: donkey anti-mouse Dylight 647 (1:200; Jackson ImmunoResearch Alexa Flour 647 donkey a-mouse #715-606-151, RRID:AB_2340866) and donkey anti-mouse FITC (1:200; Jackson ImmunoResearch FITC donkey-a-mouse 715-096-151, RRID:AB_2340796).

### Imaging

Imaging of transgenic fluorescent reporters and antibody stains within retinal sections (single plane) was performed with a Nikon Andor spinning disk confocal microscope, using a Zyla sCMOS camera and Nikon Elements software. Imaging was performed using a 40x water objective (1.15 NA). Entire retinal cryosections were stitched based on DAPI staining by using the large stitched images feature in Nikon Elements software, and were subsequently cropped to provide representative images. Image processing and analysis was performed using Nikon Elements Analysis software, and figures were assembled in PhotoShop CS6.

## Results

### Qualitative analysis of retinal electrophysiology (ERG recordings) following extensive retinal lesion

**FIGURE 1:**
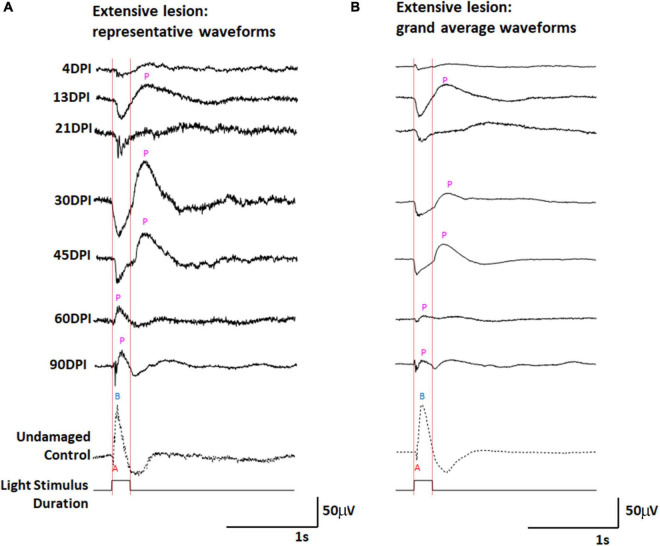
ERG waveforms in response to a 200 ms light flash, over an extended time-course of retinal regeneration following an extensive ouabain lesion. **(A)** Representative waveforms. **(B)** Grand average waveforms. A, a-wave; B, b-wave; P, post-photoreceptor response (PPR); DPI, days post-injury. Red vertical lines were added to this figure to facilitate comparison of traces in the context of light onset and offset.

We have previously documented the restoration of behavioral measures of visual recovery in zebrafish at 98 days following an extensive lesion (Sherpa et al., 2008), and at 61 days following a selective lesion (Sherpa et al., 2014). The likely limiting factor for behavioral visual recovery after an extensive lesion appeared to be growth of axons of regenerated ganglion cells toward the optic nerve head (Sherpa et al., 2014), and so we reasoned that restoration of an ERG waveform, which provides a measure of the active circuitry between photoreceptors and bipolar neurons upstream of retinal ganglion cells, may precede the behavioral recovery times. Zebrafish subjected to unilateral extensive lesioning of the retina were therefore sampled at 4, 13, 21, 30, 45, 60, and 90 DPI for retinal function through the recording of ERG responses. Representative traces (Figure 1A), and a series of grand average waveforms obtained from multiple recordings (Figure 1B) show changes in the ERG waveform and amplitudes over restoration time, in comparison to representative traces and grand average waveforms of contralateral controls. We noted considerable heterogeneity in the responses recorded from individual zebrafish retinas at many timepoints (including controls) and provide additional representative traces within Supplementary Figure 2.

The control ERG recordings display the typical characteristics of (i) a downward deflection shortly after the beginning of the 200 ms light flash, identified as the “a-wave” representing photoreceptor hyperpolarization in response to light; (ii) an upward deflection following the a-wave, that occurs during light stimulus, referred to as the “b-wave” representing ON-bipolar neurons depolarizing in response to changed input from photoreceptors; and (iii) a “photopic negative response,” a downward deflection following the b-wave, likely reflecting the physiological responses of retinal ganglion cells and Müller glia (Dowling, 1987). At 4 DPI, traces were highly irregular (Figure 1A and Supplementary Figure 2), usually low in amplitude, and the grand average showed little indication of any type of waveform deflection (Figure 1B). This sampling point likely represents a time of maximum retinal damage when any surviving neurons are inadequately functional to generate a collective response to the light flash. At 13, 21, 30, and 45 DPI, new features became apparent in the representative traces (Figure 1A and Supplementary Figures 2B-D) and the grand average waveforms (Figure 1B) of the ERG recordings obtained from regenerated retinas. Pronounced and prolonged downward deflections were observed upon light stimulus, suggesting the functional regeneration of some, or all, of the photoreceptor layer, consistent with histological observation of an outer nuclear layer (ONL) beginning at 12 DPI after an extensive lesion (Sherpa et al., 2008, 2014). Following this putative a-wave, pronounced and prolonged upward deflections were detected in several traces. For this qualitative description, we refer generically to any upward deflection that follows a downward deflection as a “post-photoreceptor response,” (PPR) because of insufficient information regarding what cellular physiology (or pathophysiology) these deflections represent in damaged and/or regenerating/regenerated retina. At 30 and 45 DPI the grand average PPR began immediately after light offset (the end of the light flash). This timing raises the possibility that the PPR visible in ERG recordings from regenerating retinas at this stage may represent an emerging d-wave, which is the depolarization of OFF-bipolar neurons in response to reduced light (Makhankov et al., 2004). In a normal, undamaged retina, the d-wave is not generally obvious after a 200 ms light flash (Figures 1A,B), likely because the contributions of electrophysiological responses of other cell types dominate the waveform (Saszik et al., 1999).

At 60 and 90 DPI, the ERG again took on new qualitative characteristics (Figures 1A,B and Supplementary Figures 2E,F). Amplitudes of deflections were apparently reduced, and the a-waves in particular began to show features more typical of a normal ERG, with a low apparent amplitude, and short duration of the negative deflection (potentially due to the continued emergence of ON-bipolar cell responses of opposite polarity). The PPRs of the grand average waveforms following the a-wave were also quite different from those observed at 13, 21, 30, and 45 DPI, with a peak taking place during, rather than following, the end of the light flash. This timing, in combination with observed changes in the a-wave, suggests that these PPRs represent an emerging b-wave, or depolarization of ON-bipolar neurons in response to the light flash.

The grand average waveforms of ERGs recorded from extensively lesioned zebrafish were scaled proportionately for each grand average, as described in Methods, allowing further inspection of the characteristics previously described (Supplementary Figure 3). Most interesting were the scaled traces of the ERGs obtained at 60 and 90 DPI, in which two PPRs were often observed (Supplementary Figure 3A). Based upon the timing of these PPRs with respect to the duration of the light flash, we speculate that the first PPRs are b-waves, representing ON-bipolar function, while the second PPRs are d-waves, representing OFF-bipolar cell function.

**FIGURE 2:**
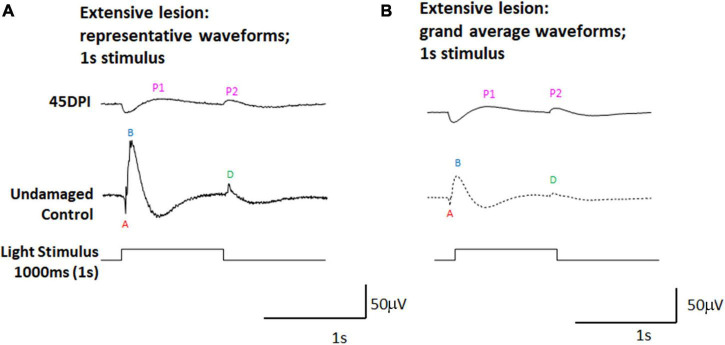
ERG waveforms in response to a 1,000 ms light flash, in undamaged controls and at 45 days post-injury (DPI) following an extensive lesion. **(A)** Representative waveforms. **(B)** Grand average waveforms. A, a-wave; B, b-wave; D, d-wave; P1 and P2, first and second post-photoreceptor responses.

To further investigate whether the dominant PPRs observed in ERGs measured at 45 DPI could represent d-waves, we subjected a separate set of adult zebrafish to extensive ouabain lesions, and recorded ERGs at 45 DPI using a 1,000 ms light flash. Longer light flashes better reveal the d-wave in undamaged retinas (Makhankov et al., 2004; Barrett et al., 2022), and we reasoned that this approach may also provide additional insights into the physiology of regenerated retinas. Representative traces (Figure 2A), and grand average waveforms obtained from these recordings (Figure 2B) indeed reveal two prominent PPRs at 45 DPI, with the first appearing shortly after light onset and possibly representing an incipient b-wave, and the second appearing at light offset and likely representing a d-wave. When scaled (Supplementary Figure 3), the apparent d-wave was similar in amplitude at 45 DPI, while the contralateral undamaged control eyes demonstrated ERGs with high amplitude b-waves and relatively lower amplitude d-waves.

### Quantitative analysis of ERG waveforms following extensive retinal lesion

**FIGURE 3:**
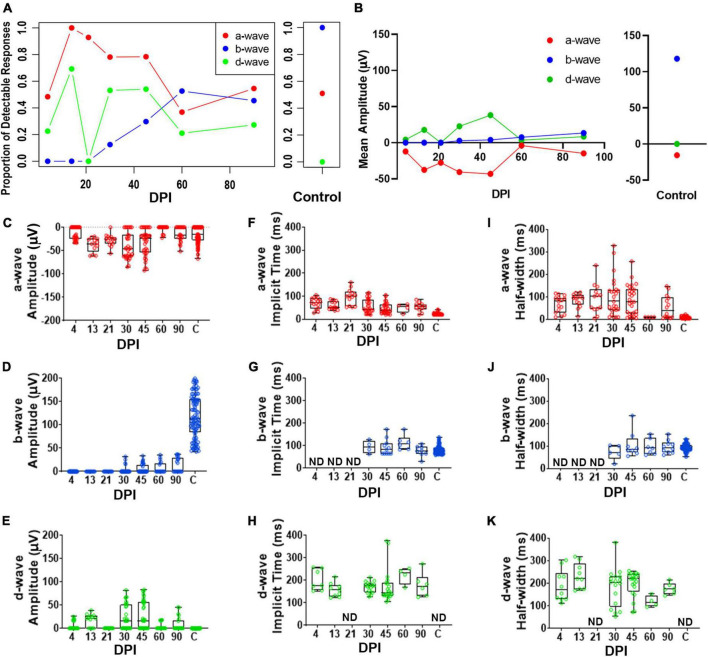
Quantitative analyses of identified a-, b-, and d-waves over a regeneration time-course following an extensive lesion. **(A)** Proportion of detectable responses (> 15 μV deflections) recorded for apparent a-waves (red filled circles; defined as negative deflections during 0-200 ms of light stimulus), b-waves (blue filled circles; positive deflections during 0-200 ms stimulus), and d-waves (green filled circles; positive deflections after end of stimulus, 200-500 ms), over regeneration time-course for extensive lesion, and for contralateral, unlesioned control eyes. **(B)** Average peak amplitudes of a-, b- and d-waves over regeneration time-course, and for contralateral, unlesioned control eyes. **(C–E)** Peak amplitudes of a-waves **(C)**, b-waves **(D)**, and d-waves **(E)**. **(F–H)** Implicit times of a-waves **(F)**, b-waves **(G)**, and d-waves **(H)**. I-K. Half-widths of a-waves **(I)**, b-waves **(J)**, and d-waves **(K)**. Each symbol represents a value for an individual eye, the horizontal band represents the median, the top and bottom of each box (where present) indicate the 75th and 25th percentiles, and the whiskers represent the maximum and minimum. DPI, days post-injury; ND, not determined.

To accomplish a quantitative analysis of ERG responses, we assigned a-, b-, and d-wave identities to individual waveforms within each recording trace for the 200 ms light flashes, based on polarity and timing with respect to the light stimulus, and measured their peak amplitudes, implicit times, and half-widths. The criteria for assignment of the wave types are described in Methods (*ERG analyses*). We noted considerable variability from fish to fish in terms of detectable measurements of these waveform components; therefore, we also determined the fraction of detectable/measurable responses (> 15 μV deflections from baseline) for each wave component isolated in the dataset. The fraction of fish with detectable a-waves changed over time following lesion and was restored to similar proportions as control fish by 90DPI (Figure 3A and Supplementary Table 1). While no control recordings had detectable d-waves, d-waves were detected in several recordings from regenerating retinas. In contrast, after lesion there were no fish producing b-wave responses through 21DPI, then the proportion contributing a detectable b-wave increased over the remaining time-course yet did not ultimately match the consistent recording of a measurable/dominant b-wave seen in all control recordings (Figure 3A and Supplementary Table 1). Overall, the proportions of fish with detectable responses indicate restoration of the waveform components on a population level over time and illustrate the heterogeneity in which these components re-emerge on an individual basis.

We found observed peak amplitudes of a-waves trended to become more negative, from 13 to 45 DPI, and then returned to amplitudes similar to those of controls at later regeneration time points (Figures 3B,C and Supplementary Table 2). Those of b-waves remained absent or low throughout the early time-course but increased in amplitude at the latter timepoints; however, b-wave amplitudes did not reach control levels even at 90 DPI (Figures 3B,D and Supplementary Table 3). An apparent d-wave was detected at 13 DPI, and d-wave amplitudes trended toward higher levels at 30 and 45 DPI. The d-wave remained measurable in some fish at later time points (60 and 90 DPI), while none of the control eyes displayed a detectable d-wave following a 200 ms light flash (Figures 3B,E and Supplementary Table 4). Over recovery time during retinal regeneration, the emerging apparent b-wave may have “masked” in part the a- and d-waves. This is consistent with our qualitative observations that show the a- and d-waves emerging first, followed by emergence of the b-wave at later timepoints. Curiously, neither b- nor d-waves could clearly be identified at 21 DPI in any of the regenerating retinas (Figures 3A,B,D,E), possibly due to continued remodeling/replacement of new and/or residual neurons; or alternatively, due to canceling contributions of de- and hyper-polarizing responses resulting in a net zero response.

Implicit times and half-widths were analyzed to determine the time-course of recovery of the waveform features. Implicit times of a- and d-waves trended highest in the middle of the time-course, while those of b-waves increased from 30 – 90 DPI (Figures 3F-K and Supplementary Tables 2–4). For some of the fish analyzed at the later timepoints, measurements were similar to those of controls, suggesting that some of the regenerated retinas had recovered the features/shape of the normal ERG waveform, with approximately similar balance among the respective components, if not the absolute amplitudes. Again, this result is consistent with qualitative observations of the waveform over time. Together, these results suggest that OFF-bipolar responses are restored earlier during regeneration compared to ON-bipolar responses, and that the balance of ON vs. OFF pathways progressively becomes more similar to control responses only at later regeneration time points.

### Qualitative analysis of retinal electrophysiology (ERG recordings) following selective retinal lesion

**FIGURE 4:**
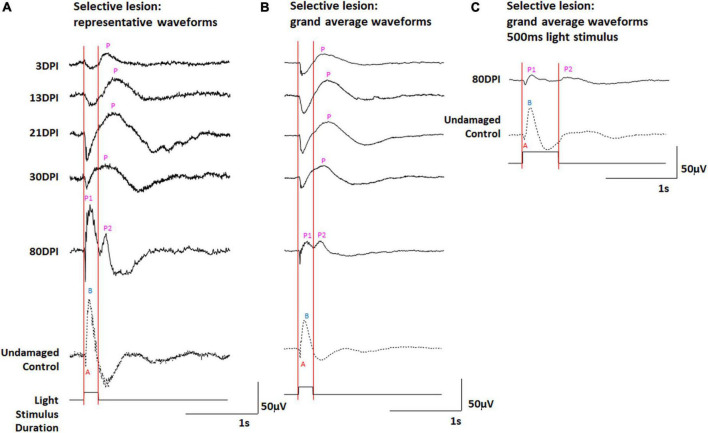
**(A,B)** ERG waveforms in response to a 200 ms light flash, over a time-course of retinal regeneration following a selective lesion. **(A)** Representative waveforms. **(B)** Grand average waveforms. **(C)** Grand average ERG waveforms in response to a 500 ms light flash, in undamaged controls and at 80 days post-injury (DPI), modified from (McGinn et al., 2018). A, a-wave; B, b-wave; P, post-photoreceptor response (PPR); P1 and P2, first and second PPRs. Red vertical lines were added to this figure to facilitate comparison of traces in the context of light onset and offset.

We have previously documented the restoration of behavioral measures of visual recovery in zebrafish at 61 days following a selective retinal lesion (Sherpa et al., 2014). In addition, we have observed normal morphologies and photoreceptor synaptic connections of a subpopulation of ON- and ON/OFF-bipolar neurons at 21 and 60 DPI (McGinn et al., 2018, 2019), and a normal ERG waveform (albeit reduced in amplitude) at 80 DPI (McGinn et al., 2018). We therefore examined the ERG recovery following selective lesion at timepoints where we have previously demonstrated synaptic connections between photoreceptors and bipolar cells in regenerated retinas, and to address the question of whether the OFF- and/or ON-bipolar circuitries become functional earlier compared to extensive lesion. Zebrafish subjected to unilateral selective lesioning of the retina were sampled at 3, 13, 21, 30, and 80 DPI for retinal function through the recording of ERGs. The ERGs recorded at 80 DPI and reported previously were repeated because the present study utilized 200 ms light flashes, and our prior study used 500 ms stimuli (McGinn et al., 2018). Representative traces (Figure 4A and Supplementary Figure 4), and a series of grand average waveforms obtained from these recordings (Figure 4B) show changes in the ERG waveform and amplitudes over recovery time, in comparison to the representative traces and grand average waveform of contralateral controls.

At 3 DPI, the ERG waveforms show a prolonged negative deflection following light onset, and a PPR that was absent or appears reduced in amplitude and highly delayed in comparison with controls (Figures 4A,B and Supplementary Figure 4). In our prior study (McGinn et al., 2018), this sampling time (3 DPI) was considered the time of maximum damage, when a photoreceptor response remains due to the “selective” nature of the damage, where photoreceptors are spared and any surviving inner retinal neurons are inadequately functional to generate a robust PPR.

At 13 and 21 DPI, representative traces and grand average waveforms still displayed a prominent a-wave, while the PPR appeared to increase in amplitude (Figures 4A,B and Supplementary Figures 4B,C). Similar to the situation with extensive lesion (at 30 and 45 DPI), these PPRs again appeared following light offset, consistent with an apparent dominating d-wave (OFF-bipolar neuron responses) rather than a b-wave (ON-bipolar neuron responses). At 30 DPI, the shape of the PPR appeared to shift toward an earlier peak (Supplementary Figure 3D), with altered latency and half-width compared to 21 DPI, possibly reflecting emerging responses of functional ON-bipolar neurons and incipient b-wave contributions to the complete waveform. As in our prior study (McGinn et al., 2018), at 80 DPI, the peak of the PPR coincided with the peak of the PPR in undamaged controls, indicating b-wave restoration (Figure 4C). In addition, a second, lower amplitude PPR was detected immediately after the 500 ms light flash (Figure 3C), although this was not made a focus of our prior study (McGinn et al., 2018). Interestingly, in the current study, using 200 ms stimuli, we observed an early and a late PPR, which could be identified in 20% of the traces (Figure 4A and Supplementary Figure 4) and were prominent in the grand average waveforms (Figure 4B). The grand average waveforms of ERGs recorded from selectively lesioned zebrafish were scaled (normalized) and revealed these two distinct PPRs again only in the 80 DPI traces (Supplementary Figure 3).

### Quantitative analysis of retinal electrophysiology (ERG recordings) following selective retinal lesion

**FIGURE 5:**
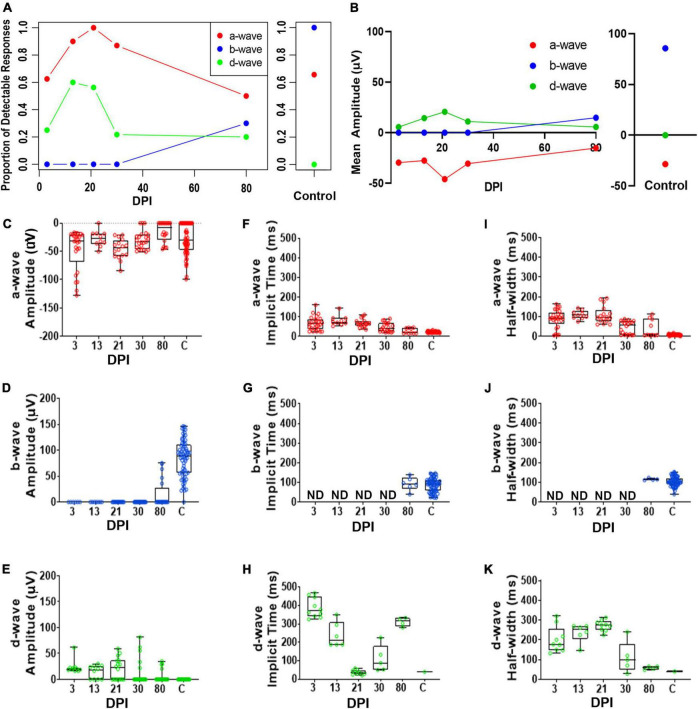
Quantitative analyses of identified a-, b-, and d-waves over a regeneration time-course following a selective lesion. **(A)** Proportion of detectable responses (> 15 μV deflections) recorded for apparent a-waves (red filled circles; defined as negative deflections during 0-100 ms of light stimulus), b-waves (blue filled circles; positive deflections during 0-100 ms stimulus), and d-waves (green filled circles; positive deflections after end of stimulus, 200-500 ms), over regeneration time-course for selective lesion, and for contralateral, unlesioned control eyes. **(B)** Average peak amplitudes of a-, b- and d-waves over regeneration time-course, and for contralateral, unlesioned control eyes. **(C–E)** Peak amplitudes of a-waves **(C)**, b-waves **(D)**, and d-waves **(E)**. **(F–H)** Implicit times of a-waves **(F)**, b-waves **(G)**, and d-waves **(H)**. **(I–K)** Half-widths of a-waves **(I)**, b-waves **(J)**, and d-waves **(K)**. Each symbol represents a value for an individual eye, the horizontal band represents the median, the top and bottom of each box (where present) indicate the 75th and 25th percentiles, and the whiskers represent the maximum and minimum. DPI, days post-injury; ND, not determined.

The same criteria were used for assignment of the wave types (described in Methods, *ERG analyses*). We again observed heterogeneity in detectable response recordings for each waveform component (Figure 5A and Supplementary Table 5). The fraction of fish with a detectable a-wave is consistent with a selective inner retinal lesion (sparing of photoreceptors) as is lack of obvious b-waves in any fish at early timepoints. Similar to retinas recovering from extensive lesion, a detectable d-wave component was dominant on a population level at early timepoints. By 80DPI, there were prominent b-waves in most fish, but this does not match the consistency in which b-waves are detected in controls. Peak amplitudes of a-waves were consistently similar to those of controls, over the entire time-course of regeneration after selective lesion (Figures 5B,C and Supplementary Table 6), again consistent with survival of photoreceptors. Peak amplitudes of b-waves remained predominantly unmeasurable throughout the time-course but emerged by the last timepoint examined (80DPI) (Figures 5B,D), when they were prominent in most traces, but not at the consistency of which b-waves were detected in controls; a number of fish at 80DPI still a exhibited a defined d-wave component. Similar to what was seen for extensive lesions, in recordings with measurable b-waves, the b-wave amplitudes did not match control levels (Figures 5B,D and Supplementary Table 7). The apparent d-waves were more obvious at earlier regeneration time points following selective lesions compared to extensive lesions, and the amplitudes of d-waves trended toward slightly higher levels until 30 DPI, and then diminished toward that of controls (Figures 5B,E and Supplementary Table 8).

Implicit times and half-widths, where detectable, also were analyzed to determine the time-course of recovery of the waveform. Implicit times of a- and d-waves trended longer early in the regeneration time-course, while b-wave features could only be analyzed when these appeared in some recordings at 80 DPI (Figures 5F–K and Supplementary Tables 6, 8). For some of the fish analyzed at the later timepoints, these measurements were similar to those of controls, suggesting that some of the regenerated retinas had recovered the main features/shape of the ERG waveform, if not the amplitudes.

### Restoration of IPL structure following extensive retinal lesion

In our prior study (McGinn et al., 2019), restoration and accumulation PKCα + bipolar neurons after a selective lesion appeared to take place from 13 to 21 DPI. This timing roughly correlates with the emergence of a PPR in several recordings after an extensive or selective lesion, with the exception that no PPR was detectable at 21 DPI after an extensive lesion (Figures 1, 3 of present study). We therefore examined the restoration of retinal neurons and synaptic layers in retinas from which ERGs were recorded, in order to correlate functional recovery to histological regeneration for retinas subjected to both extensive and selective lesions. In particular, we sought to more closely inspect (i) positions of PKCα + (ON-bipolar) axon terminals within the IPL; (ii) the overall appearance of the IPL using a general synaptic marker (anti-SV2); and (iii) the arrangement of the sublaminae of the IPL using parvalbumin as a marker, following an extensive vs. selective lesion. In adult zebrafish parvalbumin is expressed in subpopulations of amacrine cells that extend processes into the IPL in two distinct layers, visible as PARV + stripes in retinal cryosections, within sublaminae 4 (s4) and 5 (s5), both considered to be sublaminae receiving input from ON-bipolars (Yeo et al., 2009).

**FIGURE 6:**
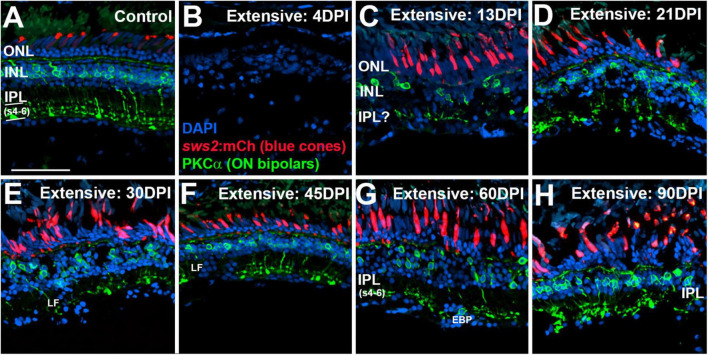
Time-course of restoration of PKCα + ON bipolar neurons and their synaptic terminals following extensive lesion. Tissues were processed for indirect immunofluorescence and imaging after ERG recordings. **(A)** Control retina displaying characteristic ON-bipolar synaptic terminals within the deep (s4-6) layers of the inner plexiform layer (IPL). **(B)** Retinal tissue at 4 days post-injury (DPI), demonstrating loss of retinal structure, and of cell types including *sws2*:mCherry + blue cones and PKCα + ON bipolar neurons. **(C–H)** Regenerating retinas at 13 **(C)**, 21 **(D)**, 30 **(E)**, 45 **(F)**, 60 **(G)**, and 90 DPI **(H)**. Some features of IPL emerge by 13 DPI, but laminar fusions (LF) remain, and PKCα + ON bipolar axon terminals are not entirely localized to IPL sublaminae s4-6. In addition, the occasional ectopic PKCα + nucleus [EBP in panel **(G)**] is found in the ganglion cell layer. ONL, outer nuclear layer; INL, inner nuclear layer; scale bar (in A, applies to all) = 50 μm.

In control retinas, synaptic terminals of PKCα + neurons were observed within the deep layers of the IPL: s6, s5, and s4 (Figure 6A), consistent with a function as ON-bipolar cells (Yazulla and Studholme, 2001). Control retinas also showed mCherry + blue cones with a regular distribution, although with occasional gaps lacking reporter expression (Figure 6A), which appears to be a feature of the *sws2:mCherry* transgenic line (Salbreux et al., 2012). At 4 DPI following an extensive ouabain lesion, staining of PKCα + material was rarely observed, and the mCherry + cones were also not evident, confirming the effectiveness of the lesion (Figure 6B). The DAPI + nuclei in 4 DPI retinas were likely a combination of Müller glia and leukocytes that survived and responded to the lesion (Sherpa et al., 2014; Mitchell et al., 2018; Barrett et al., 2022). At 13 DPI after an extensive lesion, PKCα + cell bodies were present within a poorly-defined INL, with small synaptic terminals that reached into an emerging, but also poorly-defined IPL (Figure 6C). At this time, mCherry + blue-sensitive cones were also present within an emerging ONL (Figure 6C). At 21, 30, and 45 DPI following an extensive lesion, the INL and IPL remained disorganized, but the synaptic terminals of PKCα + neurons appeared to more reliably occupy the deep layers of the IPL (Figures 6D,E,F). However, several laminar fusions remained evident, and some synaptic terminals did not reach into the deeper regions of the IPL (Figures 6D,E,F). Patterns of mCherry + cones were varied and appeared irregular or patchy, as has been shown for the teleost cone mosaic following cone regeneration (Stenkamp et al., 2001; Stenkamp and Cameron, 2002). At 60 and 90 DPI after extensive lesion, laminar organization again improved slightly (Figures 6G,H), consistent with our previous findings (Sherpa et al., 2014). The overall trajectory of changes in histological appearance and the positions of the PKCα + synaptic terminals were, in general, consistent with the findings from the ERGs, in that waveforms remained abnormal over the entire time-course, but also revealed the re-emergence of a putative b-wave at 60 and 90 DPI (Figures 1, 3), when ON-bipolar cells are seen to be more organized and stratified in the regenerated IPL.

**FIGURE 7:**
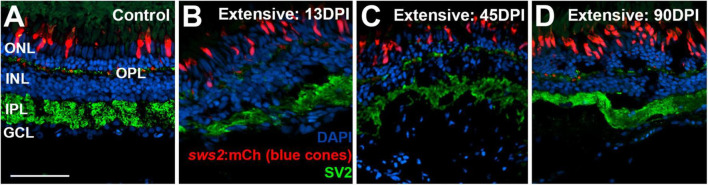
Time-course of restoration of SV2 + plexiform layers following extensive lesion. Tissues were processed for indirect immunofluorescence and imaging after ERG recordings. **(A)** Control retina showing SV2 staining within the outer plexiform layer (OPL) and inner plexiform layer (IPL). **(B–D)** Regenerating retinas at 13 **(B)**, 45 **(C)**, and 90 days post-injury (DPI) **(D)**. SV2 staining is visible but disorganized within an emerging OPL and IPL at 13 DPI **(B)**, but displays improved lamination at 45 and particularly at 90 DPI **(C,D)**. ONL, outer nuclear layer; INL, inner nuclear layer; GCL, ganglion cell layer; scale bar (in **A**, applies to all) = 50 μm.

To examine the synaptic layers, we stained retinal cryosections for the post-synaptic marker SV2. Retinas of control eyes displayed SV2 + signal within the entirety of the IPL, and along a thin band of puncta representing the OPL (Figure 7A). In retinal tissue obtained at 13 DPI following extensive lesion, puncta of SV2 staining within an emerging OPL were occasionally visible, and the IPL appeared disorganized, with variable thickness (Figure 7B). By 45 DPI, the OPL was more prominently and reliably labeled, and the IPL, while still patchy in places, took on an appearance that more closely resembled that of undamaged retina in some regions (Figure 7C). At 90 DPI, the OPL and IPL were even more consistently labeled, and with more consistent thickness (Figure 7D), again supporting the maturation of ON-bipolar dependent b-waves in the ERG traces.

**FIGURE 8:**
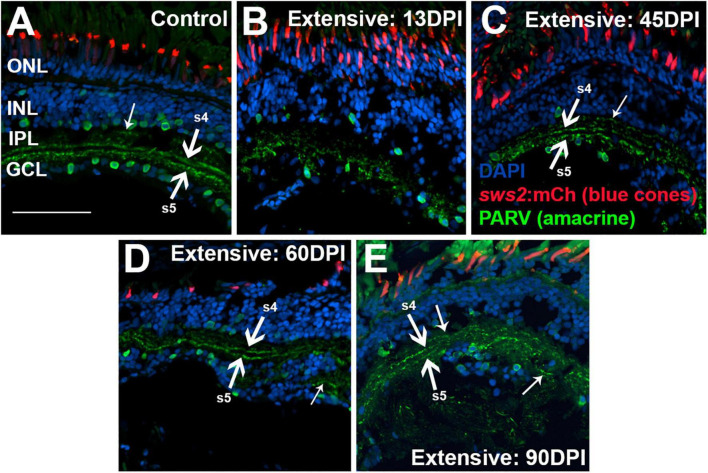
Time-course of restoration of PARV + amacrine cells and their synaptic processes following extensive lesion. Tissues were processed for indirect immunofluorescence and imaging after ERG recordings. **(A)** Control retina displaying characteristic pattern of PARV + amacrine cells within the inner nuclear layer (INL) and ganglion cell layer (GCL), and stripes of processes occupying inner plexiform layer (IPL) sublaminae s4 and s5 (thick arrows), which receive input from ON-bipolar neurons. Occasional stripes of PARV + processes were also observed within OFF sublaminae (thinner arrow). **(B–E)** Regenerating retinas at 13 **(B)**, 45 **(C)**, 60 **(D)**, and 90 days post-injury (DPI) **(E)**. PARV + sublaminae are not recognizable at 13 DPI, but can be observed at 45, 60, and 90 DPI (arrows), although these patterns are not consistent across the retina. Thick arrows again depict likely sublaminae s4 and s5, while the thinner arrows point to PARV + processes within OFF sublaminae (as in **C**), or disorganized processes with atypical lamination patterns (as in **D**). ONL, outer nuclear layer; scale bar (in A, applies to all) = 50 μm.

To examine the IPL more closely, we stained retinal cryosection for parvalbumin. Control retinas showed PARV + amacrine cell bodies within the INL and the GCL, and dendritic processes that stained two distinct stripes within the IPL, the sublaminae s4 and s5 (Yeo et al., 2009) (Figure 8A). An additional stripe within the “OFF” input sublaminae of the IPL was also occasionally weakly stained (Figure 8A). This three-stripe appearance of PARV staining has previously been noted for zebrafish larval IPL (Nevin et al., 2008), though not, to our knowledge, for adult zebrafish retina. In retinal cryosections at 13 DPI following extensive lesion, PARV + cell bodies were present within the disorganized INL and GCL, and the PARV + dendritic processes did not display a recognizable striping pattern (Figure 8B). Remarkably, retinal cryosections obtained at 45 and 60 DPI after an extensive lesion, displayed regions in which stripes of PARV + dendritic processes were evident (Figures 8C,D), supporting refinement of synaptic layers. We tentatively assigned the most recognizable stripes as potentially corresponding to sublaminae s4 and s5, as these stripes tended to reside in the deeper regions of the IPL. An additional stripe (or stripes) positioned within potential sublaminae of OFF-bipolar neurons was more rarely observed in 45 and 60 DPI retinas (Figures 8C,D). Misplaced stripes and/or puncta within the GCL were also seen (Figures 8C,D). The striped pattern of PARV + dendritic processes and INL/GCL pattern of PARV + cell bodies persisted at 90 DPI (Figure 8E). As with PKCα + synaptic terminals, the trajectory of change in PARV staining over the time-course of regeneration – suggesting the formation of ON sublaminae – was largely consistent with the ERG recordings revealing an apparent b-wave emerging around 45-60 DPI (Figures 1B, 3B and Supplementary Figure 3A).

### Restoration of IPL structure following selective retinal lesion

**FIGURE 9:**
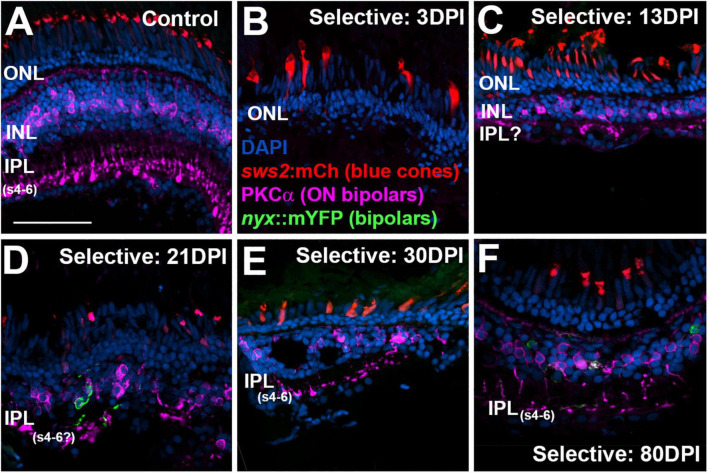
Time-course of restoration of PKCα + ON bipolar neurons and their synaptic terminals following selective lesion. Tissues were processed for indirect immunofluorescence and imaging after ERG recordings. Retinas were of zebrafish transgenic for *sws2*:mCherry (in blue cones) and *nyx*:mYFP (in a subset of bipolar neurons); in this line the mYFP is expressed in a variegated manner (Schroeter et al., 2006), therefore not all of the panels in this figure show the YFP reporter. **(A)**. Control retina displaying characteristic ON-bipolar synaptic terminals within the deep (s4-6) layers of the inner plexiform layer (IPL). **(B)**. Retinal tissue at 3 days post-injury (DPI), demonstrating loss of inner retinal structure, and particularly of PKCα + ON-bipolar neurons. Photoreceptors, including *sws2*:mCherry + blue cones, are not damaged by the selective lesion. **(C–H)**. Regenerating retinas at 13 **(C)**, 21 **(D)**, 30 **(E)**, and 80 DPI **(F)**. Some features of IPL emerge by 13 DPI **(C)**, but PKCα + ON bipolar axon terminals are not entirely localized to regions that are recognizable as sublaminae s4-6. At 21 and 30 DPI, PKCα + terminals occasionally reach what appear to be the deeper layers of the IPL **(D,E)**. At 80 DPI, PKCα + terminals are found in the deep, ON layers of the IPL **(F)**. ONL, outer nuclear layer; INL, inner nuclear layer; scale bar (in **A**, applies to all) = 50 μm.

**FIGURE 10:**
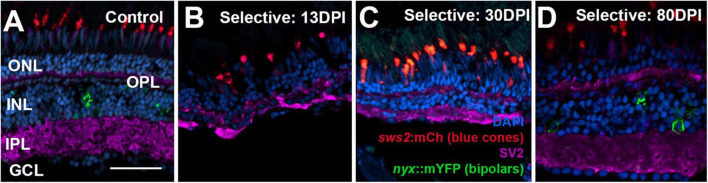
Time-course of restoration of SV2 + plexiform layers following selective lesion. Tissues were processed for indirect immunofluorescence and imaging after ERG recordings. **(A)** Control retina showing SV2 staining within the outer plexiform layer (OPL) and inner plexiform layer (IPL). **(B–D)** Regenerating retinas at 13 **(B)**, 30 **(C)**, and 80 days post-injury (DPI) **(D)**. SV2 staining is visible but disorganized within an emerging OPL and IPL at 13 DPI **(B)**, but displays improved lamination at 30 and particularly at 80 DPI **(C,D)**. ONL, outer nuclear layer; INL, inner nuclear layer; GCL, ganglion cell layer; scale bar (in **A**, applies to all) = 50 μm.

**FIGURE 11:**
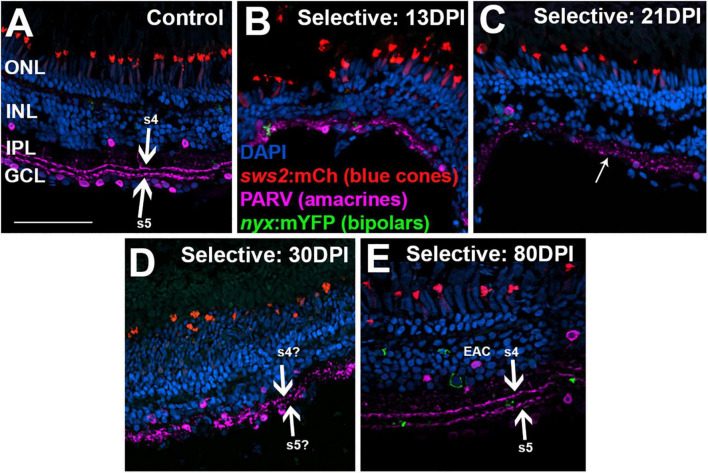
Time-course of restoration of PARV + amacrine cells and their synaptic processes following selective lesion. Tissues were processed for indirect immunofluorescence and imaging after ERG recordings. Retinas were of zebrafish transgenic for *sws2*:mCherry (in blue cones) and *nyx*:mYFP (in a subset of bipolar neurons); in this line the mYFP is expressed in a variegated manner (Schroeter et al., 2006), therefore not all of the panels in this figure show the YFP reporter. **(A)** Control retina displaying characteristic pattern of PARV + amacrine cells within the inner nuclear layer (INL) and ganglion cell layer (GCL), and stripes of processes occupying inner plexiform layer (IPL) sublaminae s4 and s5 (thick arrows), which receive input from ON-bipolar neurons. **(B–E)** Regenerating retinas at 13 **(B)**, 21 **(C)**, 30 **(D)**, and 80 days post-injury (DPI) **(E)**. PARV + sublaminae are not recognizable at 13 and 21 DPI; rather, the PARV + processes appear as disorganized puncta within the IPL (thinner arrow in **C**). Hints of these layers emerge at 30 DPI (thick arrows in **D**). At 80 DPI, the PARV + processes form two distinct stripes that may correspond with sublaminae s4 and s5 (thick arrows in **E**). PARV + cell bodies are somewhat displaced from the boundary with the IPL (EAC in **E**, ectopic amacrine cell). ONL, outer nuclear layer; scale bar (in **A**, applies to all) = 50 μm.

As we have previously demonstrated (McGinn et al., 2018, 2019), PKCα + bipolar neurons are virtually eliminated by a selective lesion, when viewed at 3 DPI, in comparison to undamaged retina (Figures 9A,B), and accumulate over the 13 – 21 DPI timeframe (Figures 9C,D) (McGinn et al., 2019). By 21 DPI some of their synaptic terminals appear localized to the deep layers of the emerging IPL (Figure 9D). The 30 DPI retinas, somewhat surprisingly, showed PKCα + neurons and terminals with little apparent improvement in appropriate patterning, although some terminals did reach the deeper layers of the IPL (Figure 9D). The PKCα + bipolar neurons at 80 DPI displayed terminals that reached the ON sublaminae of the IPL (Figure 9E), consistent with our prior evaluations of bipolar neurons following selective lesion (McGinn et al., 2018). This is also consistent with the emergence of a detectable b-wave with a near-normal waveform at this timepoint. Presence of the mYFP + bipolar neurons in the transgenic line utilized is variegated, and these cells are reduced in number in regenerated retina, as we have previously demonstrated (McGinn et al., 2019), and so they do not appear in all images in Figures 9–11.

Retinas of control eyes displayed a consistent pattern of SV2 staining in the OPL and IPL (Figure 10A). In 13 and 21 DPI retinas following selective lesion, puncta of SV2 staining within an emerging OPL were occasionally visible, and the IPL was variable in thickness, occasionally with gaps in which SV2 staining was not present (Figures 10B,C). In 30 DPI retinas, the OPL and IPL were more consistently labeled, although the IPL remained reduced in thickness (Figure 10C). At 80 DPI, the OPL and IPL displayed an SV2 labeling pattern similar to that of control retina (Figure 10D).

To better assess organization of the IPL, we again stained retinal cryosections for PARV. PARV + amacrine cell bodies appeared in 13 DPI retina in a regions apical to, basal to, and within the emerging IPL (Figures 11A,B), but PARV + synaptic processes were not clearly organized into the two distinct s4 and s5 sublaminar stripes of control retina (Figures 11A,B). At 21 DPI, PARV striping patterns stripes were still not visible; rather, the PARV + synaptic processes appeared as puncta broadly distributed within the IPL (Figure 11C). At 30 DPI, these processes again did not show clear organization into stripes (Figure 11D). At 80 DPI, the s4 and s5 sublaminar stripes were apparent, although not without some disruption (Figure 11E). The PARV + cell bodies appeared less densely distributed, particularly in the GCL, and those in the INL were in many cases displaced from their positions (seen in undamaged retinas) immediately adjacent to the IPL (Figure 11E).

## Discussion

Our studies of functional regeneration following extensive or selective retinal lesions reveal dynamic changes in ERG waveform characteristics during regeneration. While recovery of ERG responses was heterogeneous on an individual basis, the recovery showed an overall trend in which the re-emerging PPR was dominated by a presumptive d-wave early in regeneration, followed by a shift to a b-wave dominated response later in regeneration. This is notable at the population level (fish producing no responses) and at the individual level (waveform amplitude, implicit time, halfwidth). In addition, even at the final timepoints analyzed, waveforms observed in some fish retain a second PPR, suggesting the potential for persistent underlying physiological differences in comparison to undamaged controls. At the level of histology examined here, we did not note strong correlation of histological markers (such as ON-bipolar cell staining) with that of variable ERG traces within timepoints, suggesting that differences in ERG responses arise from other mechanisms beyond the staining patterns of the markers used. The simplest interpretations of these changes and heterogeneity over time during regeneration are that specific cell types and connections are regenerated, restored and/or become functional at different rates, producing changes in balance between the identified features of the waveform over time. Interestingly, in our previous study, we found that bipolar cell dendritic trees transiently decrease in spread at early timepoints (13-17 DPI) (McGinn et al., 2019), suggesting at least some degree of synaptic remodeling during rewiring. Since the ERG represents a composite of all contributions to the full field-potential response at light ON and light OFF, each with negative and/or positive polarity, the relative dominance of each component is reflected in the complete waveform. Taken together, our ERG results suggest that (1) OFF-bipolar cell functional components are restored or mature earlier compared to the ON bipolar cell components; (2) OFF-bipolar functional components are restored slightly more rapidly after selective lesion than after extensive lesion; and (3) at the latest regeneration time points examined, we observed robust ON- and OFF-bipolar cell contributions to the waveform, yet distinct abnormalities remained compared to undamaged controls, suggesting incomplete restoration and remodeling that persists in the regenerated retina, even following selective/partial lesioning. Our parallel studies of markers of ON-bipolar structural outputs, performed on the same samples from which ERGs were recorded, corroborate these findings, with appropriately stratified INL sublaminae observed only at the latest timepoints.

The sequence of functional changes we observed over time during retinal regeneration appears in some ways to recapitulate the normal developmental order observed for related functional and morphological features. This includes evidence that functional OFF-bipolar connections mature before ON-bipolar connections. This is intriguing given that re-emergence of inner retinal neuron types is overlapping, rather than sequential, in regeneration (McGinn et al., 2019). During zebrafish retinal development and maturation, ERG recordings suggest a hierarchical maturation sequence. ERG waveforms for younger zebrafish (5-24 dpf) demonstrate more robust d-waves that are distinct and readily separable from b-waves (following 560 nm light stimuli); in contrast, for older adult zebrafish the b-wave becomes so dominant that the d-wave response is masked, particularly at light stimulus durations of 200 ms or less (Saszik et al., 1999). During retinal regeneration at 30 and 45 DPI (extensive lesion) and at 21 DPI (selective lesion), we similarly observed presumptive d-waves evident at light offset, but minimal evidence of a depolarizing (b-wave) responses during the light stimulus (that is, the magnitude of the positive b-wave, if present, is mostly masked by the a-wave of opposite polarity). This interpretation was supported by longer light stimulus intervals providing further separation between ON and OFF responses. Intriguingly, later regeneration time points appeared to exhibit more balance between ON and OFF responses, yet the ratio of presumptive b-wave to d-wave amplitudes (ratio b/d) was altered in regenerated retinas compared to control/undamaged retinas. In addition, our data suggest that at mid- to later time points the a-wave latency (implicit time) is diminished, with the emergence and growing strength of the b-wave contribution. The smaller b/d-wave ratio at 90 DPI (extensive lesion) remained divergent compared to undamaged adult ERG responses. Some of the reduction in b-wave amplitude also may be related to disruption in the optical path to the retina – over the long term, ouabain-treated fish eyes can develop overgrown irises, cataracts, double lenses, and cloudy posterior and/or anterior chambers (Raymond et al., 1988; Mensinger and Powers, 1999; Cameron and Powers, 2000; Stenkamp et al., 2001; Sherpa et al., 2008). A further consideration, particularly for the extensive lesion, is that regenerated photoreceptors may not be in alignment with the pupil of the eye, and therefore less efficient at capturing incoming photons, similar to the Stiles-Crawford effect of the first kind (Nilagiri et al., 2021).

Similar to zebrafish, in developing mouse retina the expression pattern for vesicular glutamate transporter 1 demonstrates a temporal ordering of events, where OFF-bipolar circuits appear to mature prior to ON-bipolar circuits (Sherry et al., 2003). Furthermore, refinement of ON/OFF pathways with retinal maturation in mice has been shown to progress in an activity-dependent manner such that the ratio of “bistratified” vs. “mono-stratified” connections between the responding bipolar cells with retinal ganglion cells changes from ∼53% to 29% with maturation (Tian and Copenhagen, 2003). While mice show very limited capacity for retinal regeneration, selective cell ablation studies have revealed some restoration and remodeling of synaptic contacts. For example, Nemitz et al. (2021) have demonstrated differential remodeling following selective horizontal cell ablation in adults, where cone photoreceptors are able to form functional connections with OFF-bipolar cells but not invaginating-type contacts with ON-bipolar cells, suggesting that synapse formation and maintenance differs between these distinct types of retinal connections. In other words, the morphological plasticity necessary for flat photoreceptor to OFF-bipolar cell synaptogenesis/connections may be more permissive than that necessary for photoreceptor to ON-bipolar cell connections. The adult zebrafish outer retina contains both invaginating photoreceptor-bipolar synapses and “basal” (flat) connections in which the synaptic terminal lacks synaptic ribbons, which may correspond to ON- and OFF-bipolar synapses, respectively (Haverkamp et al., 2000; Tarboush et al., 2012). Further evidence from existing zebrafish mutants highlights the separable nature of ON- and OFF-pathway development and function (Allwardt et al., 2001; Jia et al., 2014; Miller et al., 2018). Thus, basal OFF-bipolar synapses with photoreceptors may regenerate and reorganize/mature more readily compared to invaginated ON-BP synapses. Our prior microanatomical studies of zebrafish cone inputs to *nyx:mYFP* + bipolar neurons (ON- and mixed ON/OFF-bipolars) indicated accurate wiring of these types of inputs at 21 DPI (McGinn et al., 2019) and 60 DPI (McGinn et al., 2018) following a selective lesion. Therefore, we were surprised to observe no measurable b-waves at 21 DPI, and some unusual responses at 80 DPI following similar lesions. Functional features of this particular circuitry may be delayed in maturation in comparison with the appearance of apparent synaptic contacts. Future studies of the ultrastructure of regenerated retinal synapses, and/or the use of pathway-specific markers of the photoreceptor-bipolar synapse may further test this hypothesis. We also acknowledge that we did not separate cone vs. rod responses in this time-course study and this will be of high interest for future work.

Besides the main ERG features probed in the current study, there are other possible contributions to the changing waveform during regeneration. Firstly, apparent slow PPRs following light OFF, which present extended (slow) implicit times at early regeneration time points, potentially may reflect electrical contributions of remodeled RPE-photoreceptor interactions (c-waves), slow photoreceptor responses, or of other retinal cells. Secondly, we sometimes observed greater than expected retinal electrical activity at early DPI timepoints following extensive lesions (compared to goldfish retinal regeneration; (Mensinger and Powers, 1999)). This unexpectedly high activity level potentially may represent incomplete tissue destruction in some eyes, and/or the reduced total amount of retina needed to be restored in the much smaller zebrafish eye to elicit a response measurable by ERG. Alternatively, these observations may parallel the atypical intrinsic hyperactivity observed in degenerating/remodeling rd1 mice retinas, which is thought to arise from remnant photoreceptors and bipolar cells, with activity (and modulation) by horizontal and amacrine cells (Borowska et al., 2011; Haq et al., 2014). Sustained activity, even in an aberrant form, has the potential to facilitate retinal neuron regeneration (Corredor and Goldberg, 2009; Lim et al., 2016; Levin et al., 2022), and we have shown that in zebrafish, surviving photoreceptors facilitate functional recovery following cytotoxic damage (Sherpa et al., 2014). Thirdly, an additional consideration for interpreting the recovery of function within the regenerating zebrafish retina is the restoration and/or remodeling of inhibitory interneuron inputs, including horizontal cells in the outer retina. Hyperpolarizing horizontal cell activity, and the timing for rewiring of horizontal cell-photoreceptor contacts, may influence a-wave amplitudes as well as post-photoreceptor output during regeneration. Furthermore, the potential delayed or incomplete restoration of invaginating contacts of horizontal cells with photoreceptors at ON-bipolar synapses may help shape the ongoing restoration of b-wave responses and the balance of ON vs. OFF pathways. Suppressive inhibitory signals (via both horizontal and amacrine cells) also are thought to mediate ON-OFF interactions and crosstalk (Popova, 2014). Restoration of indirect inhibitory pathways potentially may help explain some of the main electrophysiological features we observe during regeneration. It is also possible that the delayed maturation of the regenerated ON-bipolar neurons’ synaptic output structures (within the IPL) observed in the present study may reflect overall differentiation delays that affect numerous cell-specific functional components. Probing detailed changes in inhibitory inputs during regeneration will necessitate future physiological studies using discrete pharmacological manipulations (Wong et al., 2005).

The process of cellular retinal regeneration in zebrafish remains a topic of great interest for scientists and clinicians aiming to accomplish a similar process in human patients with loss of retinal neurons. The present study highlights the need to understand the intricacies of the re-wiring process that must also take place in order to restore useful visual function. While some waveforms remained abnormal in regenerated zebrafish retinas, and all remained low in amplitude at the longest recovery times, this level of function is sufficient to support simple reflexes (Mensinger and Powers, 1999; Sherpa et al., 2008), and more complex behaviors (Sherpa et al., 2008, 2014), indicating the adequate “success” of regenerated circuitry. It is noteworthy that most of the behavioral experimental endpoints utilized by those who study retinal regeneration in zebrafish do not allow inferences regarding the restoration of ON vs. OFF pathways. Indeed, those used in our prior studies (escape response; place preference behavior) (Sherpa et al., 2008, 2014), most likely depend primarily on the function of OFF pathways, as the fish must detect a relatively dark object against a brighter background. As the field of “mammalian retinal regeneration” matures, it will be interesting to learn whether their retinal circuitry regenerates function in a similar manner.

## Data availability statement

The original contributions presented in this study are included in the article/Supplementary material, further inquiries can be directed to the corresponding author.

## Ethics statement

This animal study was reviewed and approved by Institutional Animal Care and Use Committees of the University of Idaho and of Washington State University.

## Author contributions

LB and DM performed the experiments. All authors analyzed the data, conceived the project, and wrote the manuscript.
