# Supplementary material for: Dynamic functional and structural remodeling during retinal regeneration in zebrafish
Source: Front Mol Neurosci. 2022 Nov 30;15:1070509. doi: 10.3389/fnmol.2022.1070509 (PMC9748287; doi:10.3389/fnmol.2022.1070509)
Supplement: Supplementary file 1 [file Data_Sheet_1.PDF]

Supplemental Figures and Legends

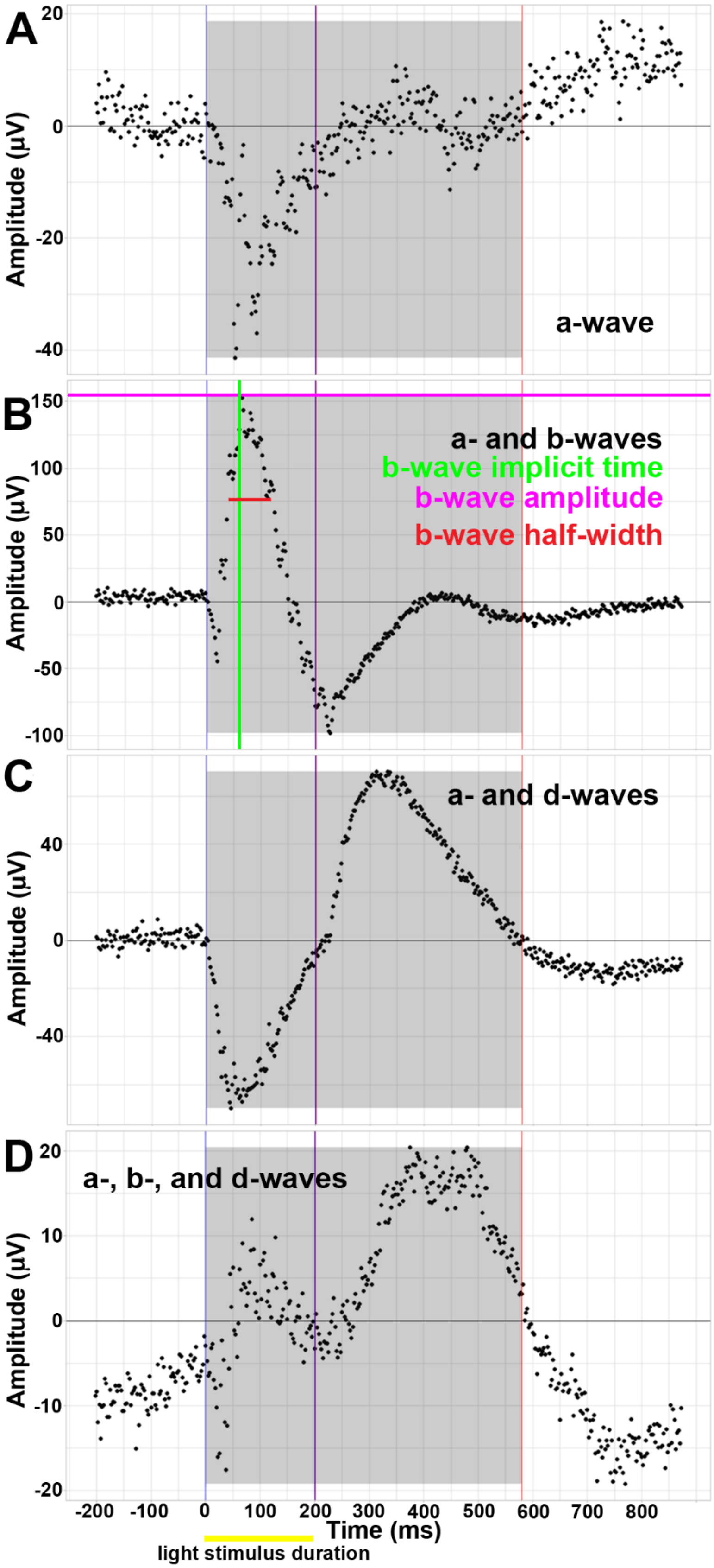

Supplemental Figure S1. Identification and quantification of apparent a-, b-, and d-wave properties in example raw ERG traces. **A.** Recording showing only an a-wave, peaking within the duration of the light flash, at an amplitude greater than 15 µV. **B.** Recording showing a- and b-waves, each peaking within the duration of the light flash, at amplitudes greater than 15 µV. Implicit time (time of maximum deflection from zero µV), amplitude (maximum deflection from zero µV), and half-width (width of wave at half-maximal amplitude) are shown for the b-wave using green, magenta, and red lines, respectively. **C.** Recording showing a- and d-waves, with the a-wave peaking during the light flash, and the d-wave peaking after the light flash. **D.** Recording showing a-, b-, and d-waves, with the a- and b-waves peaking during the light flash, and the d-wave peaking after the light flash.

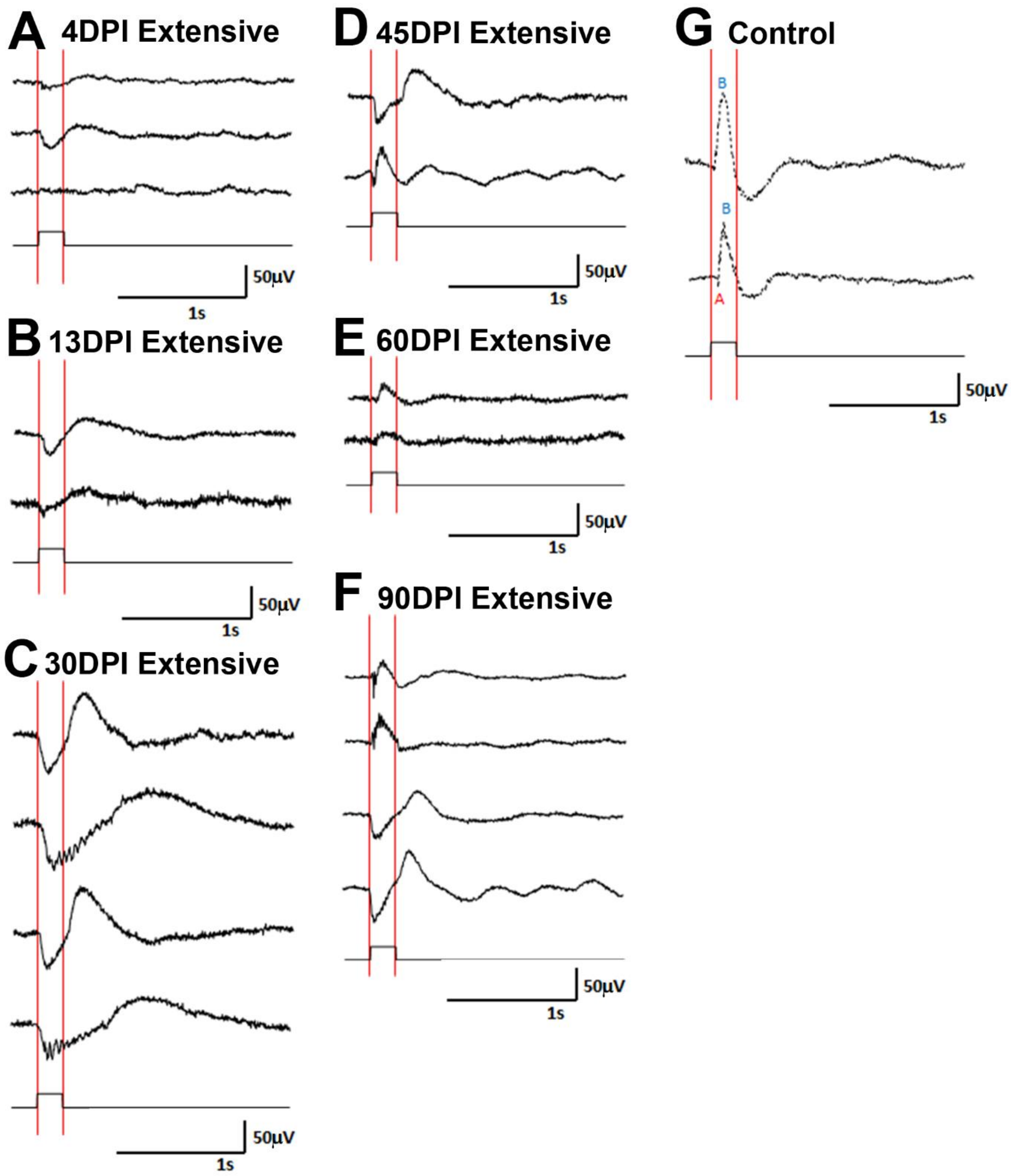

Supplemental Figure S2. Additional selected, representative ERG recordings showing heterogeneity of waveforms observed in individual zebrafish at time points following extensive lesion. The first example in each series is the same as the recording shown in Figure 1, and the other examples contained identified waves or combinations of waves (or lack of response) also frequently observed for that time point. **A.** 4 days post-injury (DPI). **B.** 13DPI. **C.** 30DPI. **D.** 45DPI. **E.** 60DPI. **F.** 90DPI. **G.** Control. Red vertical lines were added to this figure to facilitate comparison of traces in the context of light onset and offset.

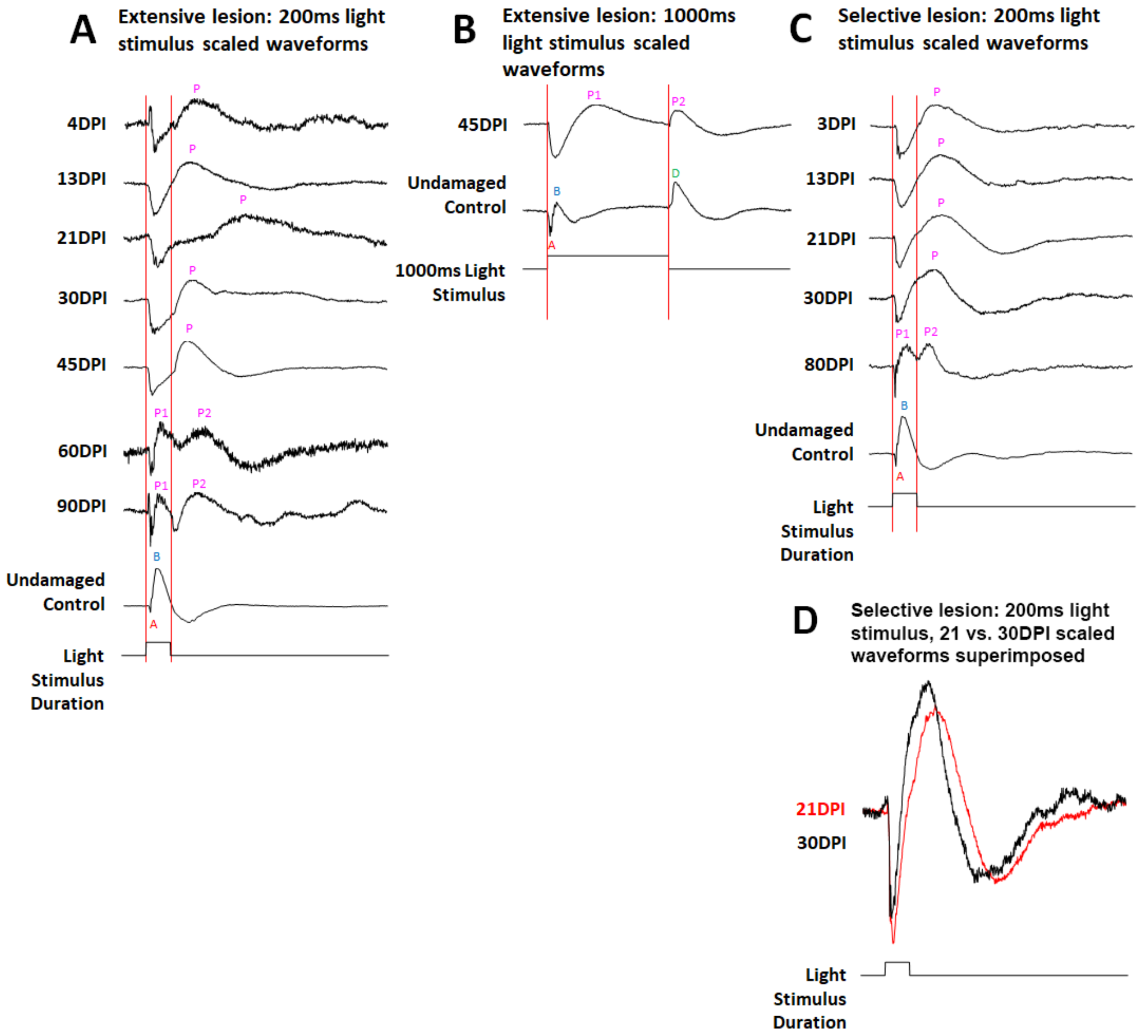

Supplemental Figure S3. ERG waveforms (grand averages) scaled to the maximum (100%) and minimum (0%) amplitudes for each grand average. **A.** Extensive lesion, 200 ms light stimulus. **B.** Extensive lesion, 1000 ms (1s) light stimulus. **C.** Selective lesion, 200 ms light stimulus. DPI, days post-injury; P, post-photoreceptor response (PPR); P1, PPR during light flash; P2, PPR after light flash; A, a-wave; B, b-wave; D, d-wave. Red vertical lines were added to this figure to facilitate comparison of traces in the context of light onset and offset. D. Selective lesion, 200 ms light stimulus, display of scaled grand average waveforms of 21DPI and 30DPI recordings superimposed, with initial baseline recordings aligned, showing the leftward shift and altered half-width in PPR.

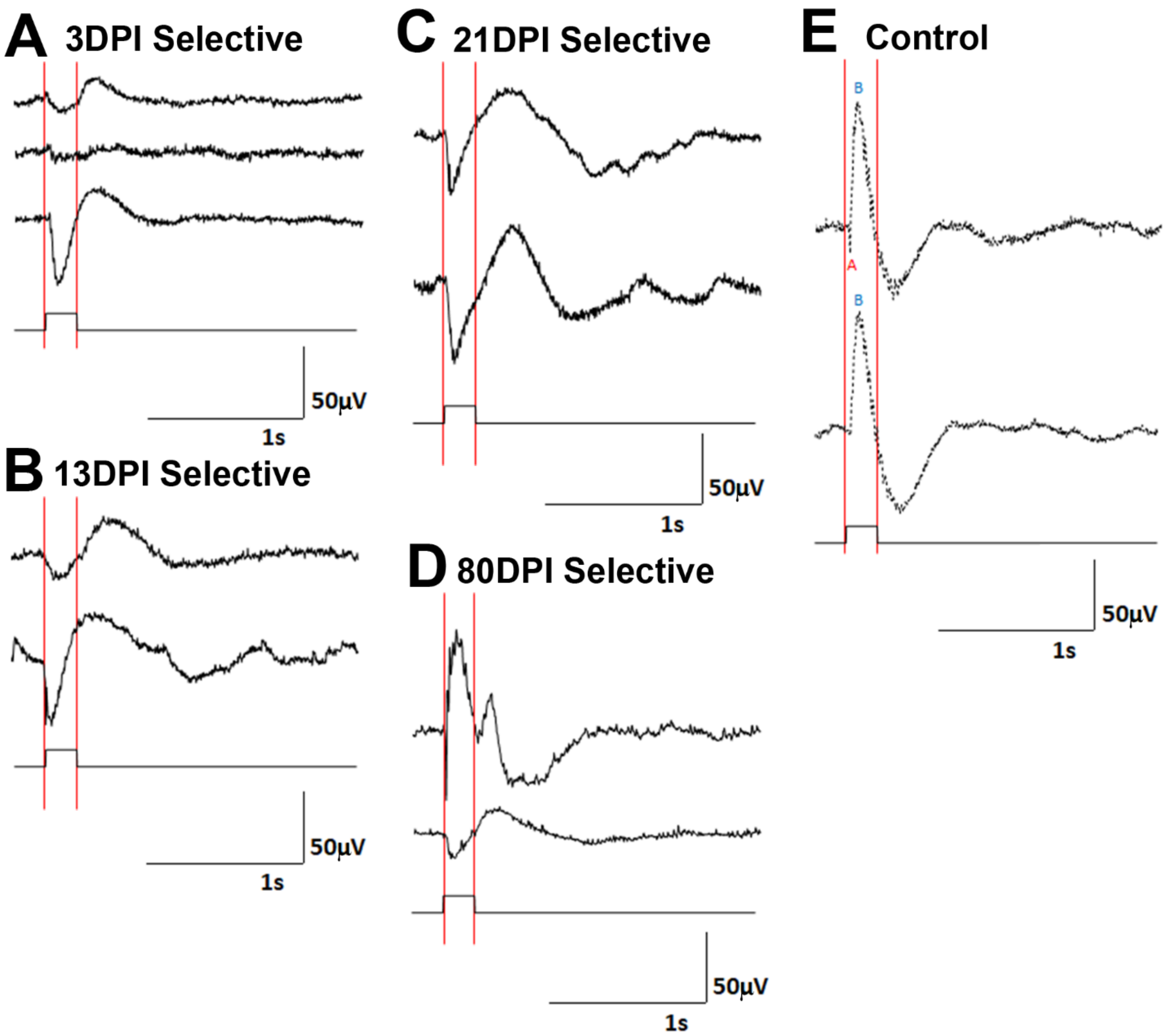

Supplemental Figure S4. Additional selected, representative ERG recordings showing heterogeneity of waveforms observed in individual zebrafish at time points following selective lesion. The first example in each series is the same as the recording shown in Figure 4, and the other examples contained identified waves or combinations of waves (or lack of response) also frequently observed for that time point. **A.** 3 days post-injury (DPI). **B.** 13DPI. **C.** 21DPI. **D.** 80DPI. **E.** Control. Red vertical lines were added to this figure to facilitate comparison of traces in the context of light onset and offset.
