# Supplementary material for: Dynamic functional and structural remodeling during retinal regeneration in zebrafish
Source: Front Mol Neurosci. 2022 Nov 30;15:1070509. doi: 10.3389/fnmol.2022.1070509 (PMC9748287; doi:10.3389/fnmol.2022.1070509)
Supplement: Supplementary file 2 [file Data_Sheet_2.PDF]

Supplemental Tables

Supplemental Table S1. Statistical analyses of proportion of detectable responses following extensive lesion.

**a-wave, X^2 p = 8.699e-07; post-hoc pairwise proportion tests:**							
	Control	4DPI	13DPI	21DPI	30DPI	45DPI	60DPI
4DPI	1.00						
13DPI	0.0212	0.0295					
21DPI	0.0707	0.0835	1.00				
30DPI	0.1212	0.2279	0.8644	1.00			
45DPI	0.796	0.1692	0.8644	1.00	1.00		
60DPI	1.00	1.00	0.0081	0.0295	0.0707	0.0511	
90DPI	1.00	1.00	0.0805	0.2279	0.8644	0.7634	1.00
**b-wave, X^2 p = 2.2e-16; post-hoc pairwise proportion tests:**							
	Control	4DPI	13DPI	21DPI	30DPI	45DPI	60DPI
4DPI	<2e-16						
13DPI	<2e-16	-					
21DPI	<2e-16	-	-				
30DPI	<2e-16	0.29356	0.66490	0.66490			
45DPI	<2e-16	0.01462	0.20813	0.19119	0.5014		
60DPI	9.5e-12	0.00011	0.02249	0.01722	0.02474	0.50140	
90DPI	8.4e-14	0.00052	0.04427	0.03593	0.66423	0.66490	0.66490
**d-wave, X^2 p = 8.99e-07; post-hoc pairwise proportion tests:**							
	Control	4DPI	13DPI	21DPI	30DPI	45DPI	60DPI
4DPI	1.9e-05						
13DPI	<2e-16	0.0601					
21DPI	-	0.4771	0.0029				
30DPI	8.3e-4	0.1885	1.00	0.0113			
45DPI	2.6e-14	0.1319	1.00	0.0084	1.00		
60DPI	5.4e-05	1.00	0.1092	0.4771	0.2933	0.2358	
90DPI	1.5e-06	1.00	0.2151	0.3554	0.4771	0.4511	1.00

Supplemental Table S2. Statistical analyses of a-wave parameters following extensive lesion.

a-wave amplitude, extensive lesion, p-values (Kruskal-Wallis with Dunn post-hoc)							
	Control	4DPI	13DPI	21DPI	30DPI	45DPI	60DPI
4DPI	4.0e-06						
13DPI	0.001	4.7e-09					
21DPI	0.03	1.0e-06	0.40				
30DPI	4.2e-05	1.7e-12	0.67	0.59			
45DPI	0.003	3.1e-10	0.24	1.0e-06	0.34		
60DPI	0.03	0.23	1.7e-05	7.0e-04	1.4e-06	4.2e-05	
90DPI	0.9	0.001	0.004	0.06	0.002	0.03	0.09
**a-wave implicit time, extensive lesion, p-values (Kruskal-Wallis with Dunn post-hoc)**							
	Control	4DPI	13DPI	21DPI	30DPI	45DPI	60DPI
4DPI	2.8e-08						
13DPI	1.0e-06	0.75					
21DPI	3.1e-10	0.07	0.48				
30DPI	7.1e-08	0.43	0.70	0.14			
45DPI	4.0e-06	0.12	0.33	0.02	0.53		
60DPI	0.046	0.53	0.71	0.36	0.86	0.85	
90DPI	5.0e-04	0.33	0.53	0.12	0.75	0.85	0.93
**a-wave half-width, extensive lesion, p-values (Kruskal-Wallis with Dunn post-hoc)**							
	Control	4DPI	13DPI	21DPI	30DPI	45DPI	60DPI
4DPI	6.0e-06						
13DPI	5.7e-07	0.73					
21DPI	1.0e-06	0.83	0.93				
30DPI	2.4e-10	0.73	0.94	0.93			
45DPI	2.4e-10	0.83	0.82	0.93	0.83		
60DPI	0.73	0.13	0.07	0.07	0.06	0.07	
90DPI	0.002	0.51	0.23	0.28	0.17	0.25	0.36

Supplemental Table S3. Statistical analyses of b-wave parameters following extensive lesion.

**b-wave amplitude, extensive lesion, p-values (Kruskal-Wallis with Dunn post-hoc)**							
	Control	4DPI	13DPI	21DPI	30DPI	45DPI	60DPI
4DPI	2.9e-12						
13DPI	1.3e-11	1.0					
21DPI	2.9e-12	1.0	1.0				
30DPI	2.7e-20	0.73	0.78	0.78			
45DPI	8.6e-19	0.44	0.53	0.53	0.44		
60DPI	4.2e-11	0.46	0.53	0.53	0.64	0.99	
90DPI	7.1e-10	0.15	0.29	0.29	0.31	0.56	0.68
**b-wave implicit time, extensive lesion, p-values (Kruskal-Wallis)**							
Null hypothesis not rejected.							
**b-wave half-width, extensive lesion, p-values (Kruskal-Wallis)**							
Null hypothesis not rejected.							

Supplemental Table S4. Statistical analyses of d-wave parameters following extensive lesion.

**d-wave amplitude, extensive lesion, p-values (Kruskal-Wallis with Dunn post-hoc)**							
	Control	4DPI	13DPI	21DPI	30DPI	45DPI	60DPI
	0.04						
13DPI	7.5e-07	0.002					
21DPI	1.0	0.20	2.3e-04				
30DPI	4.8e-09	0.004	0.44	3.3e-04			
45DPI	8.4e-11	0.001	0.55	1.4e-04	0.84		
60DPI	0.14	0.90	0.004	0.31	0.008	0.003	
90DPI	0.02	0.66	0.01	0.11	0.03	0.02	0.61
**d-wave implicit time, extensive lesion, p-values (Kruskal-Wallis)**							
Null hypothesis not rejected.							
**b-wave half-width, extensive lesion, p-values (Kruskal-Wallis)**							
Null hypothesis not rejected.							

Supplemental Table S5. Statistical analyses of proportion of detectable responses following selective lesion.

**a-wave, X^2 p = 0.0004242; post-hoc pairwise proportion tests:**					
	Control	3DPI	13DPI	21DPI	30DPI
3DPI	1.00				
13DPI	0.850	0.768			
21DPI	0.076	0.059	0.985		
30DPI	0.473	0.386	1.00	0.850	
80DPI	0.985	1.00	0.353	0.013	0.102
**b-wave, X^2 p = 8.669e-07; post-hoc pairwise proportion tests:**					
	Control	3DPI	13DPI	21DPI	30DPI
3DPI	<2e-16				
13DPI	<2e-16	-			
21DPI	<2e-16	-	-		
30DPI	<2e-16	-	-	-	
80DPI	1.1e-12	0.001	0.053	0.033	0.014
**d-wave, X^2 p = 8.669e-07; post-hoc pairwise proportion tests:**					
	Control	3DPI	13DPI	21DPI	30DPI
3DPI	0.00034				
13DPI	1.5e-09	0.24444			
21DPI	2.7e-09	0.24444	1.00		
30DPI	0.00146	1.00	0.24444	0.24444	
80DPI	0.99271	1.00	0.24444	0.24444	1.00

Supplemental Table S6. Statistical analyses of a-wave parameters following selective lesion.

**a-wave amplitude, selective lesion, p-values (Kruskal-Wallis with Dunn post-hoc)**					
	Control	3DPI	13DPI	21DPI	30DPI
3DPI	0.48				
13DPI	0.95	0.64			
21DPI	0.02	0.008	0.14		
30DPI	0.54	0.25	0.71	0.14	
80DPI	0.09	0.28	0.25	0.001	0.06
**a-wave implicit time, selective lesion, p-values (Kruskal-Wallis with Dunn post-hoc)**					
	Control	3DPI	13DPI	21DPI	30DPI
3DPI	1.4e-08				
13DPI	3.7e-07	0.33			
21DPI	4.6e-08	0.66	0.60		
30DPI	3.2e-04	0.12	0.03	0.06	
80DPI	0.96	1.1e-04	7.6e-05	8.4e-05	0.02
**a-wave half-width, selective lesion, p-values (Kruskal-Wallis with Dunn post-hoc)**					
	Control	3DPI	13DPI	21DPI	30DPI
3DPI	6.4-09				
13DPI	2.4e-07	0.30			
21DPI	6.0e-09	0.43	0.69		
30DPI	0.008	0.02	0.005	0.005	
80DPI	0.09	6.4e-09	0.005	0.005	0.69

Supplemental Table S7. Statistical analyses of b-wave parameters following selective lesion.

**b-wave amplitude, selective lesion, p-values (Kruskal-Wallis with Dunn post-hoc)**					
	Control	3DPI	13DPI	21DPI	30DPI
3DPI	3.5e-21				
13DPI	2.4e-08	1.0			
21DPI	1.2e-11	1.0	1.0		
30DPI	5.5e-15	1.0	1.0	1.0	
80DPI	8.8e-10	0.55	0.64	0.59	0.58
**b-wave implicit time, selective lesion, p-values (Kruskal-Wallis)**					
Null hypothesis not rejected					
**b-wave half-width, selective lesion, p-values (Kruskal-Wallis)**					
Null hypothesis not rejected					

Supplemental Table S8. Statistical analyses of d-wave parameters following selective lesion.

**d-wave amplitude, selective lesion, p-values (Kruskal-Wallis with Dunn post-hoc)**					
	Control	3DPI	13DPI	21DPI	30DPI
3DPI	0.014				
13DPI	2.0e-04	0.03			
21DPI	2.0e-06	0.01	0.91		
30DPI	0.03	0.91	0.04	0.02	
80DPI	0.07	0.91	0.03	0.01	0.91
**d-wave implicit time, selective lesion, p-values (Kruskal-Wallis with Dunn post-hoc)**					
	Control	3DPI	13DPI	21DPI	30DPI
3DPI	-				
13DPI	-	0.12			
21DPI	-	0.15	0.75		
30DPI	-	0.32	0.04	0.04	
80DPI	-	0.008	0.17	0.12	0.003
**d-wave half-width, selective lesion, p-values (Kruskal-Wallis with Dunn post-hoc)**					
	Control	3DPI	13DPI	21DPI	30DPI
3DPI	-				
13DPI	-	0.53			
21DPI	-	0.09	0.32		
30DPI	-	0.16	0.09	0.006	
80DPI	-	0.051	0.03	0.001	0.51
